# Assessment of USP17 and TPT1 Expression in Primary and Matched Metastatic Tissue of Patients with High-Grade Serous Ovarian Carcinoma and the Relationship to Distant Metastasis

**DOI:** 10.3390/diagnostics16172704

**Published:** 2026-08-25

**Authors:** Ezgi Bilicen, Hasan Bahadır Saatlı, Meral Koyuncuoğlu, Erkan Çağlıyan

**Affiliations:** 1 Department of Obstetrics and Gynecology, Uşak Training and Research Hospital, Uşak 64100, Türkiye; 2 Department of Obstetrics and Gynecology, Faculty of Medicine, Dokuz Eylul University, İzmir 35340, Türkiye; hb.saatli@gmail.com (H.B.S.); erkcagliyan@yahoo.com (E.Ç.); 3 Department of Pathology, Faculty of Medicine, Dokuz Eylul University, İzmir 35340, Türkiye; mrl.koyuncuoglu@gmail.com

**Keywords:** high-grade serous ovarian carcinoma, metastasis, ubiquitin-proteasome pathway, ubiquitin-specific protease 17, translationally controlled tumor protein 1, deubiquitinating enzymes

## Abstract

**Objectives:** Deubiquitinating enzymes have been increasingly implicated in tumor progression and metastasis. Ubiquitin-specific protease 17 (USP17) and translationally controlled tumor protein 1 (TPT1) are associated with cell proliferation, migration, and cancer progression; however, their differential expression in matched primary and metastatic tissues of high-grade serous ovarian carcinoma (HGSC) has not been well defined. This study aimed to compare USP17 and TPT1 expression between matched primary and metastatic HGSC tissues and to explore their association with metastatic disease. **Methods:** This retrospective study included 33 patients diagnosed with HGSC. Immunohistochemical analysis of USP17 and TPT1 expression was performed using tissue microarrays (TMA) constructed from matched primary ovarian tumor tissues and corresponding metastatic tissues. USP17 expression was evaluated using a semi-quantitative composite immunohistochemical scoring system, while TPT1 expression was assessed using a semi-quantitative immunoreactivity index. Paired statistical analyses were applied to compare expression patterns between primary and metastatic tissues. **Results:** Primary and metastatic USP17 expression levels showed a positive correlation (Spearman’s rho = 0.349, *p* = 0.047) whereas paired comparison showed no significant difference between the two sites (*p* = 0.438). In contrast, TPT1 expression showed no statistically significant correlation (Spearman’s rho = 0.285, *p* = 0.107) or paired comparison showed no difference (*p* = 0.107) between matched primary and metastatic tissues. USP17 and TPT1 expression levels were significantly positively correlated in both primary (Spearman’s rho = 0.406, *p* = 0.019) and matched metastatic tissues (Spearman’s rho = 0.351, *p* = 0.045). Neither USP17 nor TPT1 expression showed a significant association with International Federation of Gynecology and Obstetrics (FIGO) stage, lymph node involvement, or receipt of neoadjuvant chemotherapy (NACT). **Conclusions:** These findings indicate a positive correlation between USP17 expression in paired primary and metastatic HGSC tissues without significant changes in overall expression levels, though causality cannot be inferred. Conversely, TPT1 expression showed no significant correlation or site-specific variation between matched tumor tissues. Further prospective and functional studies are required to validate these findings and clarify their biological significance.

## 1. Introduction

Ovarian cancer is one of the leading causes of gynecologic cancer-related mortality worldwide [1]. Approximately 95% of ovarian malignancies are of epithelial origin, with high-grade serous ovarian carcinoma (HGSC) representing the most common and lethal subtype compared with non-serous histological subtypes [2,3,4]. Distant metastasis is present in 61% of patients at the time of diagnosis [5]. Current management of advanced HGSC includes either primary debulking surgery followed by platinum-based adjuvant chemotherapy or, in selected patients, neoadjuvant chemotherapy (NACT) followed by interval debulking surgery. The standard systemic treatment for HGSC for the last 30 years is platinum-based chemotherapy, in which most patients respond first and then develop resistance [4]. Currently, the International Federation of Gynecology and Obstetrics (FIGO) staging system is used for staging ovarian cancer.

Ubiquitination is the main regulatory process involved in DNA repair and the regulation of the cell cycle. Many studies were performed to investigate the link between ubiquitination and tumorigenesis [6]. Dysregulation of the ubiquitin–proteasome system has been implicated in tumorigenesis and cancer progression [7,8]. Deconjugation of ubiquitin is carried out by deubiquitinating enzymes (DUBs), which are also essential for cell functions and tumorigenesis [9].

Ubiquitin-specific protease 17 (USP17), also designated DUB3, is encoded by a tandemly repeated gene family embedded within a copy-number-variable region of the genome [10]. USP17 regulates the intracellular localization of Ras and other small GTPases that control cell proliferation and migration, and has been implicated in tumor growth and metastasis [11,12].

Also, USP17 has been linked to inflammatory signaling in the tumor microenvironment: IL-6 induces its expression, which in turn stabilizes Snail1 and sustains epithelial–mesenchymal transition, and elevated USP17 expression has been associated with recurrence and distant metastasis in solid tumors [12,13].

Translationally controlled tumor protein 1 (TPT1) is a highly conserved, ubiquitously expressed protein that is overexpressed in a wide range of tumors and has been implicated in cancer progression [14]. TPT1 is involved in many functions in cells, such as microtubule stabilization, calcium binding, anti-apoptotic effect (acting antagonistically to p53), regulation of cell growth, and DNA repair during the cell cycle [14,15]. TPT1 has a negative effect on p53 and increases its degradation [16]. Overexpression of TPT1 has been reported in several malignancies and is associated with tumor progression, chemoresistance and poor prognosis, including in epithelial ovarian cancer [17,18].

Although increased expression of USP17 and TPT1 has been reported in various cancers, data directly comparing their expression in matched primary and metastatic HGSC tissues are limited. Therefore, this study aimed to evaluate USP17 and TPT1 expression in matched primary and metastatic tissues from patients with HGSC and to investigate their association with metastatic disease and clinicopathological parameters.

## 2. Materials and Methods

This retrospective study included patients who underwent surgery for adnexal masses at Dokuz Eylül University Faculty of Medicine, Department of Obstetrics and Gynecology, between June 2013 and June 2017. A total of 33 patients with histologically confirmed HGSC and complete clinicopathological data, who had available paired primary ovarian and matched metastatic tissue specimens suitable for immunohistochemical evaluation and a determinable pathological FIGO stage, were included in the study irrespective of NACT status. Tumor stage was assigned according to the 2014 FIGO staging system [19]. Clinicopathological data, including FIGO stage, were obtained from the patients’ medical records.

Patients were excluded if they had a histological diagnosis other than HGSC, including low-grade serous carcinoma, a diagnosis of ovarian metastasis, had insufficient tissue for immunohistochemical evaluation, could not have pathological FIGO staging reliably determined because of extensive treatment-related changes following NACT, or had incomplete clinicopathological or follow-up data.

Patients were classified according to the initial treatment strategy as those undergoing primary debulking surgery or those receiving NACT followed by interval debulking surgery. For patients receiving NACT, paired primary ovarian and matched metastatic tissue specimens were obtained from interval debulking surgery and were processed using the same pathological protocol as those from patients undergoing primary surgery.

For metastatic tissue sampling, lymph node metastases were preferentially selected whenever representative tumor tissue was available. When an adequate metastatic focus was not identified in the lymph node specimen, metastatic omental tissue was used instead. Because the objective of the study was to compare primary and metastatic lesions, metastatic specimens were analyzed collectively regardless of their anatomical site.

**Figure 1:**
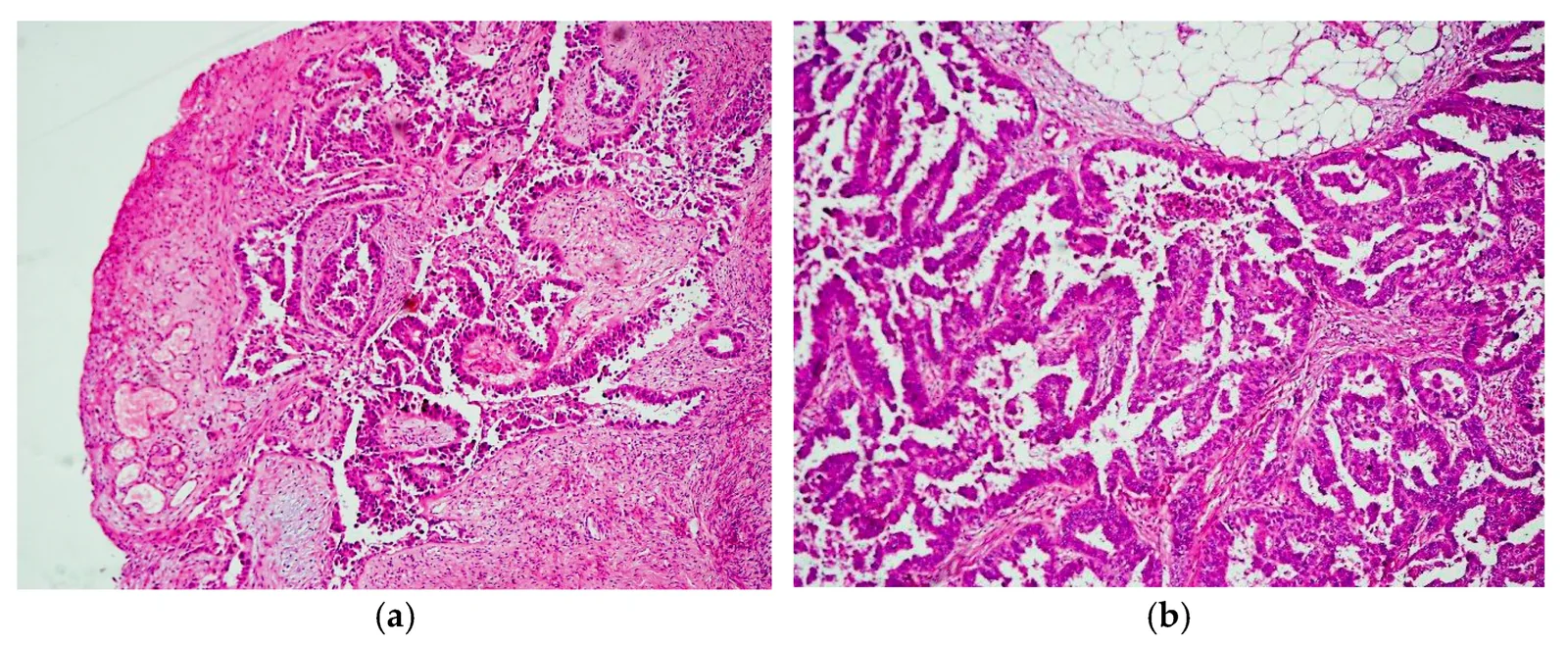
Representative H&E-stained sections of matched primary and metastatic HGSC. (**a**) Primary tumor tissue. (**b**) Matched metastatic tumor tissue. Magnification ×100.

Archived Hematoxylin and Eosin (H&E)-stained sections from both primary and metastatic tumor blocks were reviewed by an experienced gynecologic pathologist to confirm the diagnosis of HGSC and to identify representative tumor areas for tissue microarray (TMA) construction. Representative H&E-stained sections of primary and matched metastatic HGSC tissues are shown in Figure 1. Additional immunohistochemical staining with diagnostic markers such as WT1 and p53 was not performed because the study was based on archived cases with previously established histopathological diagnoses, which were reviewed on H&E sections before inclusion. Representative areas containing viable HGSC were marked, while regions of necrosis, hemorrhage, and processing artifact were avoided. For each patient, one representative tissue core was obtained from the primary tumor and one representative tissue core from the corresponding matched metastatic lesion, resulting in two tissue cores per case. Missing, substantially damaged, nonrepresentative, or tumor-deficient cores were excluded from evaluation. Only cases with evaluable matched primary and metastatic cores were retained for paired analysis. Tissue cores (0.6 mm in diameter) were created from donor paraffin-embedded tissue blocks using a drill fitted with a stainless-steel capillary tube. Each capillary tube containing a tissue core was detached from the drill, and the core was subsequently extracted using a stainless-steel wire and transferred into the corresponding hole of the recipient paraffin block. By repeating these steps, all tissue cores were precisely inserted into the recipient block containing 20 preformed holes arranged in a 4 × 5 array to construct the TMA blocks.

### 2.1. Immunohistochemical Staining

Preliminary optimization experiments were performed to determine the optimal antibody dilution and primary antibody incubation conditions before immunohistochemical staining. Stomach and bladder tissues were used as positive controls for USP17 and TPT1, respectively. Tissue sections mounted on poly-L-lysine-coated slides were incubated at 50 °C for 12 h to ensure adequate slide adhesion and drying before automated staining. This step was performed solely as a pre-treatment for slide preparation and was independent of the automated deparaffinization process carried out by the VENTANA Benchmark XT staining system. Immunohistochemical staining was performed using the VENTANA Benchmark XT automated staining system (Ventana Medical Systems, Tucson, AZ, USA). Based on the optimized laboratory protocol, rabbit polyclonal primary antibodies against USP17 (anti-USP17L2, Cat No: HPA045642, Triple A Polyclonals; Atlas Antibodies, Stockholm, Sweden) and TPT1 (anti-TPT1, Cat No: HPA039437, Triple A Polyclonals; Atlas Antibodies, Stockholm, Sweden) were applied at a dilution of 1:50 and incubated for 72 min. Following immunohistochemical staining, the slides were dehydrated through graded ethanol solutions (70%, 80%, 95%, and absolute ethanol), cleared in xylene, and mounted with DPX mounting medium (Entellan^®^, Merck, Darmstadt, Germany).

All immunohistochemically stained slides were evaluated in a blinded manner by a single experienced gynecologic pathologist. USP17 and TPT1 immunoreactivity were assessed as described below.

### 2.2. Evaluation of USP17 Immunoreactivity

USP17 immunoreactivity was evaluated using a semi-quantitative composite scoring system, integrating the proportion of positive tumor cells and staining intensity into a composite score (0–6). The proportion of positive tumor cells was assigned a score ranging from 0 to 3 according to the extent of immunoreactive tumor cells. Cytoplasmic staining intensity was scored as 0 (no staining), 1 (weak), 2 (moderate), or 3 (strong). The proportion and intensity scores were summed to generate a composite score ranging from 0 to 6, in line with previously reported immunohistochemical assessment of DUB3/USP17 in epithelial ovarian cancer [20]. For categorical analyses, cases with composite scores of 0–2 were classified as negative, whereas those with scores of 3–6 were classified as positive for USP17 expression.

### 2.3. Evaluation of TPT1 Immunoreactivity

TPT1 immunoreactivity was evaluated according to the semi-quantitative expression index described by Chen et al., allowing direct comparison with previously published studies investigating TPT1 immunohistochemical expression [21]. Staining intensity was scored on a scale of 0–3 (0 = no staining, 1 = weak, 2 = moderate, and 3 = strong), while the percentage of positively stained tumor area was recorded. The expression index was calculated using the formula: Expression Index = *I* + 10 × *P*, where *I* represents the staining intensity score and *P* represents the proportion of positively stained tumor area. Based on the calculated expression index, TPT1 expression was categorized as no expression (score = 0), weak expression (scores = 1–4), moderate expression (scores = 5–8), or strong expression (scores = 9–12).

### 2.4. Statistical Analysis

Statistical analysis of the data obtained during the study process was performed in the IBM SPSS Statistics version 27.0 computer package program (IBM Corp., Armonk, NY, USA). Semi-quantitative immunohistochemical expression scores are presented as median (25th–75th percentile), whereas continuous variables such as age are presented as mean ± standard deviation. Comparisons among FIGO stages were performed using the Kruskal–Wallis test, while comparisons between patients who received NACT and those who underwent primary surgery were performed using the Mann–Whitney U test. Differences between matched primary and metastatic tissue expression scores were evaluated using the Wilcoxon signed-rank test, and correlations between paired expression scores were assessed using Spearman’s rank correlation coefficient. Categorical paired comparisons between primary and metastatic tissues were performed using the marginal homogeneity test for TPT1 expression categories and McNemar’s test for dichotomized USP17 expression categories. Vital status was assessed at a single predefined time point using the available medical records. A *p*-value < 0.05 was considered statistically significant.

## 3. Results

The patients ranged in age from 25 to 76 years, with a mean age of 57.69 ± 13.45 years. Twenty-five patients (75.8%) underwent primary debulking surgery, whereas eight patients (24.2%) received NACT followed by interval debulking surgery. Postoperative adjuvant chemotherapy was administered to 30 patients (90.9%), and 9.1% (*n* = 3) could not receive adjuvant chemotherapy due to their poor clinical condition. The study included 21.2% (*n* = 7) with stage IIIB, 63.6% (*n* = 21) with stage IIIC, and 15.2% (*n* = 5) with stage IV. At the time of follow-up, 12 patients (36.4%) had died, whereas 21 (63.6%) were alive. Mortality was 28.6% among patients with stage IIIB or IIIC disease and 80% among those with stage IV disease.

**Figure 2:**
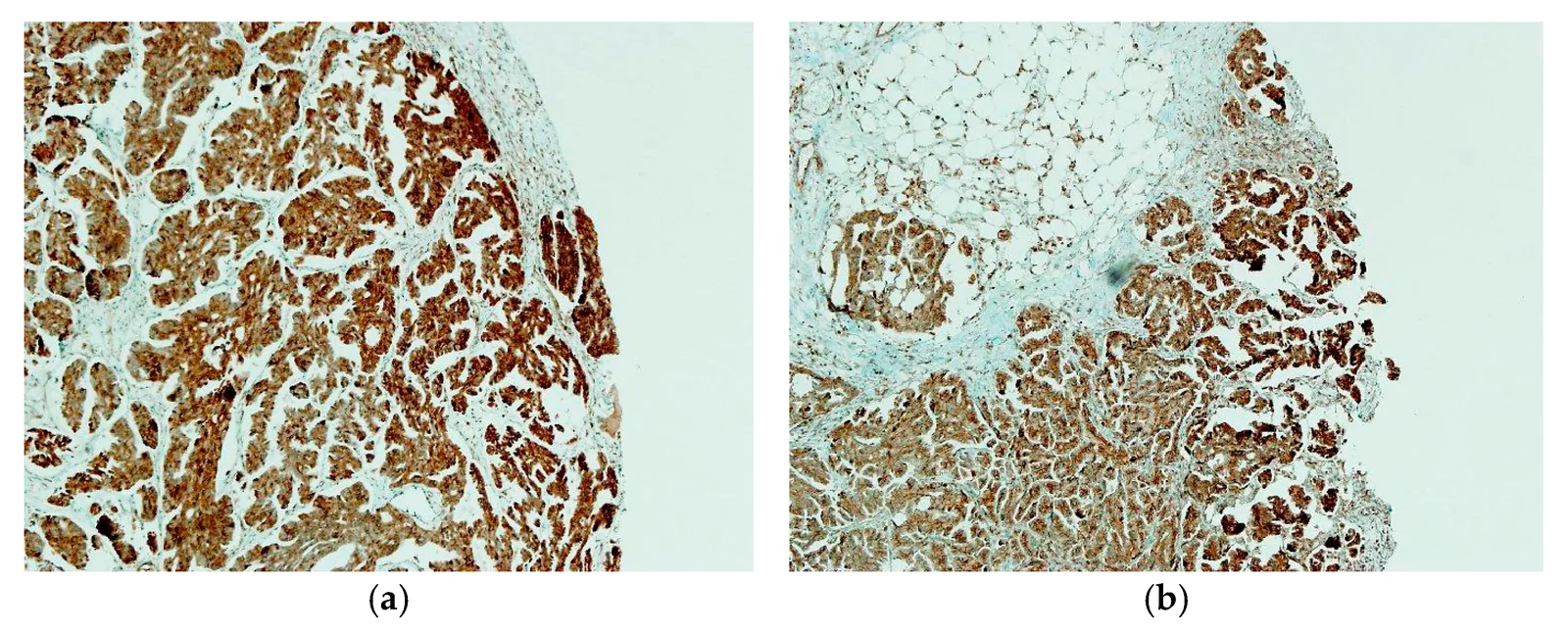
Representative immunohistochemical staining of USP17 in matched primary and metastatic HGSC tissues. (**a**) Primary tumor tissue. (**b**) Matched metastatic tumor tissue. The images are representative examples of USP17 immunoreactivity and are not intended to illustrate the complete range of the semi-quantitative scoring system. Magnification ×100.

Primary and metastatic USP17 expression levels showed a positive correlation (Spearman’s rho = 0.349, *p* = 0.047). However, no statistically significant difference was observed between matched primary and metastatic USP17 expression levels (Wilcoxon signed-rank test, *p* = 0.438). These findings suggest that tumors with higher USP17 expression in the primary lesion tended to exhibit higher expression in the corresponding metastatic tissue. Representative immunohistochemical staining patterns of USP17 expression in primary and matched metastatic tissues are shown in Figure 2.

**Figure 3:**
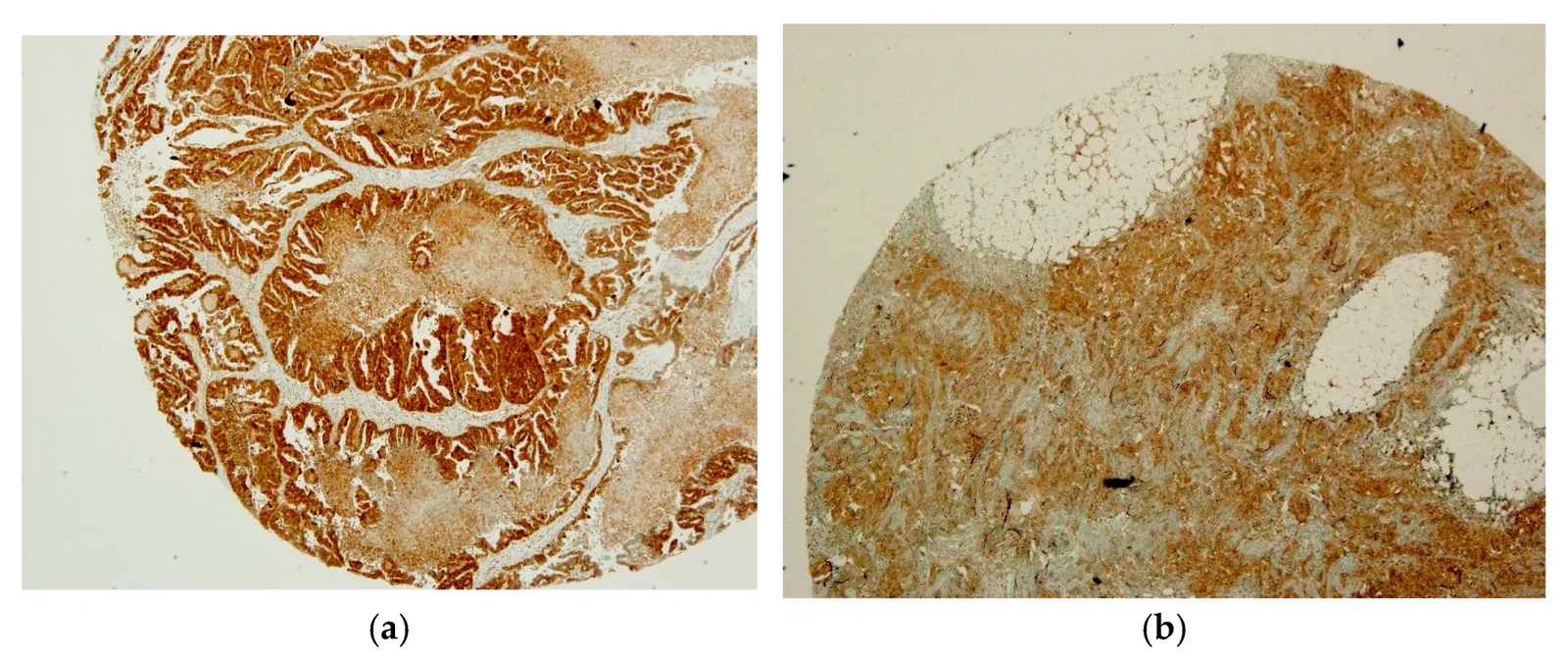
Representative immunohistochemical staining of TPT1 in matched primary and metastatic HGSC tissues. (**a**) Primary tumor tissue. (**b**) Matched metastatic tumor tissue. The images are representative examples of TPT1 immunoreactivity and are not intended to illustrate the complete range of the semi-quantitative scoring system used for evaluation. Magnification ×40.

No significant correlation was observed between primary and metastatic TPT1 expression levels (Spearman’s rho = 0.285, *p* = 0.107). Similarly, matched primary and metastatic TPT1 expression levels did not differ significantly (Wilcoxon signed-rank test, *p* = 0.166) (Table 1). Representative immunohistochemical staining patterns of TPT1 expression in primary and matched metastatic tissues are shown in Figure 3.

For both primary and matched metastatic tissues, neither USP17 nor TPT1 expression levels showed significant associations with FIGO stage, lymph node metastasis, or NACT status. Additionally, although higher USP17 expression was more frequently noted in patients who died during the follow-up period, this trend did not reach statistical significance.

A statistically significant moderate positive correlation was observed between USP17 and TPT1 expression levels in primary tumor tissues (Spearman’s rho = 0.406, *p* = 0.019). Similarly, metastatic USP17 and TPT1 expression levels showed a statistically significant positive correlation (Spearman’s rho = 0.351, *p* = 0.045).

The expression levels of both markers were evaluated according to the disease stage. No statistically significant differences were observed among FIGO stages for either USP17 or TPT1 expression (Table 2).

Median USP17 and TPT1 expression scores were lower in patients who received NACT than in those who underwent primary debulking surgery, both in primary and matched metastatic tissues. However, none of these differences reached statistical significance (Table 3).

No statistically significant difference was observed in the categorical distribution of TPT1 expression between matched primary and metastatic tissues (marginal homogeneity test, *p* = 0.149) (Table 4).

Similarly, no statistically significant difference was observed in the categorical distribution of USP17 expression between matched primary and metastatic tissues (McNemar test, *p* = 0.344) (Table 5).

## 4. Discussion

In the present study, we evaluated the expression profiles of USP17 and TPT1 in paired primary and metastatic HGSC tissues to investigate their potential roles in tumor progression and metastatic dissemination. Our paired-tissue design provides a distinct methodological advantage, allowing a direct intra-patient comparison that reduces confounding genetic and environmental backgrounds. A notable finding of our study was that primary and metastatic USP17 expression levels showed a positive correlation. However, no statistically significant difference was observed between matched primary and metastatic USP17 expression levels. Although these findings do not support a site-specific difference in USP17 expression, they suggest that USP17 may reflect biological characteristics that are maintained during metastatic dissemination, highlighting its potential relevance in HGSC progression.

Metastatic dissemination remains the principal cause of mortality in HGSC, highlighting the need to identify biomarkers associated with metastatic progression [4]. Deubiquitinating enzymes (DUBs) are key regulators of ubiquitin-mediated signaling and have emerged as important modulators of tumor development, progression, and therapeutic response. Consequently, DUBs have attracted increasing attention as potential therapeutic targets in cancer because of their diverse oncogenic and tumor-suppressive functions [8,22]. USP17 (also designated DUB3) is among the better-characterized members of this family. It regulates the subcellular trafficking of small GTPases and is required for chemokine-directed cell motility [11], controls G1–S cell-cycle progression [23], and stabilizes the oncogenic phosphatase CDC25A [24]. Loss of USP17 function in experimental systems consistently impairs proliferation, migration and invasion [25]. Notably, its effect on Ras signaling is substrate-selective: USP17 restricts N-Ras membrane trafficking and activation while leaving K-Ras unaffected [26], indicating that its influence on GTPase-dependent pathways cannot be reduced to a simple gain-or loss-of-function model.

Clinical data have generally linked higher USP17 expression to more aggressive disease. In non-small cell lung cancer (NSCLC), elevated USP17 expression was associated with recurrence, distant metastasis and shorter survival, although independent prognostic significance was not established [12]. In ovarian cancer, the first immunohistochemical study of DUB3/USP17 reported positive staining in 58.9% of epithelial ovarian carcinomas with little or no immunoreactivity in normal ovarian tissue; expression correlated with lymph node involvement and clinical stage, independently predicted overall survival, and its knockdown reduced proliferation and promoted apoptosis in ovarian cancer cells [20]. More recently, DUB3 has been implicated in platinum resistance in ovarian cancer through MGMT-driven stabilization of MCL1 [27].

Against this background, our findings extend the existing evidence by demonstrating that USP17 expression is positively correlated between matched primary and metastatic HGSC tissues, despite the absence of a significant difference in expression levels between the two sites. These findings suggest that USP17 expression may be preserved during metastatic progression rather than undergoing substantial site-specific changes.

Two considerations argue that this apparent discrepancy is more likely to reflect biology and methodology than contradiction. First, the phase of metastatic progression matters. Using transcriptomic profiling of matched primary tumors, malignant ascites and peritoneal implants from patients with HGSC, Yildirim and colleagues found that USP17 family members were predominantly upregulated in ascites-derived malignant cells rather than in established peritoneal implants, and proposed that this axis is engaged chiefly during the epithelial–mesenchymal transition (EMT) phase of dissemination [28]. Established metastatic deposits are, by definition, past that phase and have largely undergone mesenchymal–epithelial reversion; a return towards or below primary-tumor expression levels would therefore be expected rather than surprising. Second, transcript abundance and protein abundance are not interchangeable. Yildirim and colleagues measured mRNA, whereas the present study evaluated protein expression by immunohistochemistry. Previous studies have shown that USP17 function is regulated at multiple biological levels and is closely linked to cell-cycle regulation and protein stability, which may contribute to discrepancies between mRNA and protein expression [23,24].

Taken together, these observations suggest that USP17 is dynamically regulated across the successive phases of HGSC progression rather than being uniformly up- or downregulated, and they underline the value of integrating transcriptomic and protein-level data when interpreting biomarker behavior during metastasis.

A dynamic, EMT-associated pattern of USP17 activity is also consistent with the pathways in which the enzyme participates. Inflammatory signaling converges on this axis: IL-6 induces DUB3 expression, which in turn deubiquitinates and stabilizes Snail1, thereby sustaining EMT and promoting invasion and metastasis [13]. DUB3 activity is further coupled to cell-cycle control through CDK4/6-dependent activation, again converging on SNAIL1 [29], and to inflammatory and stemness programs in the tumor microenvironment [30]. Although HGSC is not classically regarded as a RAS-driven malignancy [31], alterations in RAS-related signaling networks contribute to ovarian cancer progression and to metastatic adaptation [32]. A model in which USP17 is transiently required at the point of dissemination, and is subsequently downregulated once metastatic colonies are established, would accommodate both the published transcriptomic data and our protein-level findings, although it remains a hypothesis that this study was not designed to test.

Regarding TPT1, this highly conserved and ubiquitously expressed protein contributes to cell proliferation, survival, microtubule dynamics and the cellular stress response [14,33,34,35]. Its role in malignancy has been documented across several tumor types: TPT1 knockdown reduced proliferation, migration and invasion and suppressed tumor growth and liver metastasis in colon adenocarcinoma models [36], and siRNA-mediated silencing induced caspase-dependent apoptosis in prostate cancer cells [37]. TPT1 also engages the p53 axis reciprocally—it promotes p53 degradation and attenuates p53-mediated apoptosis [16], while p53 transcriptionally induces TPT1 under oxidative stress, thereby promoting cell survival [21]. Interestingly, that same study found a positive correlation between p53 and TPT1 expression in normal lung tissue but not in lung tumors, suggesting that the regulatory relationship is disrupted during malignant transformation—an observation of particular relevance to HGSC, in which *TP53* mutation is near-universal [31]. In epithelial ovarian cancer specifically, higher TPT1 expression has been associated with advanced disease, greater proliferative activity, reduced cisplatin sensitivity and poorer survival [18].

In our cohort, however, TPT1 expression did not differ between matched primary and metastatic tissue (*p* = 0.107) and showed no association with clinicopathological parameters. These results do not argue against a role for TPT1 in ovarian cancer biology; rather, they suggest that within HGSC its expression reflects general programs of tumor-cell maintenance and survival, which are equally required at primary and metastatic sites, rather than a site-specific metastatic phenotype. Consistent with this interpretation, USP17 and TPT1 expression levels showed a significant positive correlation in both primary (Spearman’s rho = 0.406, *p* = 0.019) and matched metastatic tissues (Spearman’s rho = 0.351, *p* = 0.045), which may indicate shared upstream regulation of proliferative and survival programs without implying a common role in metastatic transition.

Neither USP17 nor TPT1 expression was significantly associated with FIGO stage, lymph node involvement, or receipt of NACT. Median USP17 and TPT1 scores were consistently lower in patients treated with NACT, in both primary and metastatic tissue, but none of these comparisons reached statistical significance. Given that only eight patients received NACT, and that the FIGO subgroups similarly contained few patients, these analyses were underpowered; the absence of statistical significance should therefore not be read as evidence of absence of an effect. This caveat is particularly relevant for the NACT comparison, since chemotherapy-induced modulation of DUB expression is biologically plausible and DUB3 has been directly linked to platinum resistance in ovarian cancer [27].

From a translational standpoint, the ubiquitin–proteasome system, and DUBs in particular, continue to attract interest as druggable targets, and several USP inhibitors have progressed through preclinical development [38,39,40,41]. Our data are exploratory and do not support the use of USP17 as a clinical biomarker or therapeutic target in HGSC. What they do indicate is that USP17 protein expression is not static across the metastatic cascade in this tumor type, and that studies sampling only primary tumors may not capture its behavior.

This study has several limitations that should be considered when interpreting the findings. First, this was a retrospective, single-center study with a relatively small sample size, which may limit the generalizability of the results. In particular, subgroup analyses according to FIGO stage, NACT status, and survival included relatively few patients and may therefore have been underpowered to detect statistically significant differences; accordingly, non-significant findings should be interpreted with caution. Second, TMA technology evaluates representative tumor areas rather than the entire tissue volume. Although representative regions were carefully selected on H&E-stained sections by an expert gynecologic pathologist, using a single tissue core per lesion may not fully capture intratumoral biomarker heterogeneity. Third, metastatic lesions from different anatomical sites were analyzed collectively, as the primary aim was to compare matched primary and metastatic tissues rather than to assess inter-site variability. Fourth, all immunohistochemical evaluations were performed by a single blinded pathologist, precluding the assessment of interobserver variability. Fifth, although all archived H&E slides were re-evaluated to confirm the original diagnosis before TMA construction, additional immunohistochemical confirmation using diagnostic markers such as WT1 and p53 was not part of the study protocol. Sixth, BRCA mutation status was unavailable because routine testing was not consistently established at our institution during the 2013–2017 study period; thus, its potential influence on USP17 and TPT1 expression could not be evaluated. Finally, survival status was assessed at a single predefined time point rather than through standardized longitudinal follow-up, precluding calculation of the median follow-up duration. Prospective, multicenter studies with larger cohorts, standardized follow-up, whole-slide assessment, and paired transcriptomic and proteomic profiling across the full metastatic cascade—primary tumor, ascites, and established metastatic deposits—will be required to determine whether USP17 plays a genuine stage-dependent role in HGSC progression. Accordingly, the present findings should be considered hypothesis-generating and require external validation. In the future, larger independent cohorts should be examined to validate the present findings. Based on the observed correlation between matched primary and metastatic USP17 expression (Spearman’s rho = 0.349), an a priori power analysis indicated that approximately 62 patients would be required to detect a correlation of this magnitude with 80% statistical power at a two-sided α level of 0.05.

Finally, photomicrographs representing the complete spectrum of immunohistochemical staining categories could not be retrieved from the available archival material; therefore, the figures are limited to representative examples, while the complete categorical distributions are reported quantitatively in the corresponding tables.

## 5. Conclusions

In conclusion, the present study demonstrates a positive correlation regarding USP17 expression between matched primary and metastatic tissues of patients with HGSC. These findings provide preliminary evidence supporting a possible association between USP17 expression and the metastatic process; however, they do not establish causality or immediate clinical applicability. In contrast, TPT1 expression showed no significant correlation between matched primary and metastatic sites, suggesting that its levels may not reflect metastatic-site-specific biological dynamics in HGSC. Larger prospective studies and functional investigations are required to validate these observations and to clarify the biological role of USP17 in HGSC progression.

## Figures and Tables

**Table 1:** Correlation of USP17 and TPT1 expression levels between matched primary ovarian tumors and corresponding metastatic tissues.

Variables	Correlation Coefficient	Spearman’s Rho *p* Value
USP17	0.349	0.047
TPT1	0.285	0.107

**Table 2:** Comparison of USP17 and TPT1 immunohistochemical expression scores across FIGO stages.

Expressions/Stage	Stage IIIB	Stage IIIC	Stage IV	*p* Value
USP17 expression in primary tissue	4 (3.5–6)	4 (3.5–6)	5 (3.5–6)	0.686
USP17 expression in metastatic tissue	4 (3–6)	4 (2–6)	5 (3.5–6)	0.622
TPT1 expression in primary tissue	6 (4–12)	10 (4–12)	8 (5–11)	0.969
TPT1 expression in metastatic tissue	8 (3–10)	8 (4–10)	10 (8–11)	0.213

Data are presented as median (25th–75th percentile). *p* values were calculated using the Kruskal–Wallis test. USP17 and TPT1 expressions were categorized according to the predefined immunohistochemical scoring criteria described in the Section 2.

**Table 3:** Comparison of USP17 and TPT1 expressions of patients who received NACT (*n* = 8) and those who did not (*n* = 25).

Expression Score	NACT (No) (*n* = 25)	NACT (Yes) (*n* = 8)	*p* Value
USP17 in primary tissue	5 (4–6)	3.5 (3–4)	0.062
USP17 in metastatic tissue	4 (3–6)	3.5 (2–5.75)	0.293
TPT1 in primary tissue	10 (4.5–12)	6 (2.5–9.5)	0.117
TPT1 in metastatic tissue	8 (4–10)	4 (3–8)	0.071

NACT: Neoadjuvant chemotherapy. Data are presented as median (25th–75th percentile). *p* values were calculated using the Mann–Whitney U test. USP17 and TPT1 expressions were categorized according to the predefined immunohistochemical scoring criteria described in the Section 2.

**Table 4:** Categorization of TPT1 expressions.

Categories for TPT1	TPT1 in Primary Tissue	TPT1 in Metastatic Tissue	*p* Value
0 (No expression)	3.0% (*n* = 1)	9.1% (*n* = 3)	0.149
1 (1–4 low expression)	24.2% (*n* = 8)	30.3% (*n* = 10)	
2 (5–8 intermediate expression)	24.2% (*n* = 8)	24.2% (*n* = 8)	
3 (9–12 high expression)	48.4% (*n* = 16)	36.4% (*n* = 12)	

Paired categorical distributions between primary and metastatic tissues were compared using the marginal homogeneity test.

**Table 5:** Categorization for USP17 expressions.

Categories for USP17	USP17 in Primary Tissue	USP17 in Metastatic Tissue	*p* Value
Negative (0–2)	9.0% (*n* = 3)	21.2% (*n* = 7)	0.344
Positive (3–6)	90.9% (*n* = 30)	78.8% (*n* = 26)	

*p* values were calculated using McNemar’s test.

## Data Availability

The datasets generated and/or analyzed during the current study are not publicly available due to patient privacy and ethical restrictions, but are available from the corresponding author on reasonable request.

## Abbreviations

The following abbreviations are used in this manuscript:

DUBs	Deubiquitinating enzymes
EMT	Epithelial–mesenchymal transition
FIGO	International Federation of Gynecology and Obstetrics
H&E	Hematoxylin and Eosin
HGSC	High-grade serous ovarian carcinoma
IHC	Immunohistochemistry
NACT	Neoadjuvant chemotherapy
NSCLC	Non-small cell lung cancer
TMA	Tissue microarray
TPT1	Translationally controlled tumor protein 1
USP	Ubiquitin-specific protease
USP17	Ubiquitin-specific protease 17
